# Prevalence and associated prenatal and perinatal risk factors for oropharyngeal dysphagia in high-risk neonates in a South African hospital

**DOI:** 10.4102/sajcd.v66i1.637

**Published:** 2019-11-21

**Authors:** Melissa A. Da Costa, Esedra Krüger, Alta Kritzinger, Marien A. Graham

**Affiliations:** 1 Department of Speech-Language Pathology and Audiology, University of Pretoria, Pretoria, South Africa; 2 Department of Science, Mathematics and Technology Education, University of Pretoria, Pretoria, South Africa

**Keywords:** Neonatal oropharyngeal dysphagia, high-risk neonate, prevalence, associated risks, Neonatal Feeding Assessment Scale

## Abstract

**Background:**

The prevalence of neonatal oropharyngeal dysphagia (OPD) in high-risk infants in lower-middle-income countries is unknown.

**Objectives:**

To determine the prevalence and associated risks for OPD in high-risk neonates in order to allow timely intervention for OPD, minimising negative outcomes.

**Method:**

A prospective cross-sectional observational study was conducted in an urban hospital in South Africa. Clinical feeding assessments were conducted using the Neonatal Feeding Assessment Scale with all available neonates in neonatal care.

**Results:**

The sample of 81 high-risk neonates (mean chronological age = 11.7 days; standard deviation = 15.6 days) had been feeding orally for 2 days and were approaching discharge. Fifty-two participants (64.2%) had OPD. Risks likely associated with OPD included breech presentation, septicaemia and other infections, spending more than 1 day on a warm table or incubator, neurological conditions, prenatal exposure to maternal smoking, siblings with mental or neurological disability, participants with congenital disorders, preterm birth (< 37 weeks), low birth weight (< 2500 g), or retinopathy of prematurity.

**Conclusion:**

An unexpected high prevalence of OPD was found in neonates already deemed ready for oral feeding and approaching discharge. Timely early involvement of the Speech-Language Therapists (SLTs) in decision-making about feeding readiness may prevent serious complications of neonatal OPD. Findings may inform South African neonatal clinicians. The study provides motivation for early intervention from SLTs before the infant and mother are discharged from high care and dispersed to communities where intervention services may be scarce.

## Introduction

Neonatal oropharyngeal dysphagia (OPD) is now considered a global concern, with negative implications for growth, general development, and independent feeding skills affecting the youngest population of high-risk infants (Cafferkey & Harrington, 2018; Rawool, 2017; Shaker, 2017). This high-risk neonatal population may comprise infants who are born preterm with low birth weight (LBW), or who have congenital conditions, neurological compromise, septicaemia, or recovering post-surgery (Jadcherla, 2016). Neurological disturbances such as neonatal encephalopathy, hypoxic-ischaemic encephalopathy, and intraventricular haemorrhage are increasingly associated with OPD in neonates (Faqeih et al., 2018; Krüger, Kritzinger, & Pottas, 2017). Associated medical conditions of OPD may include necrotising enterocolitis, respiratory distress syndrome, tachypnoea or apnoea, patent ductus arteriosus as well as bronchopulmonary dysplasia (Jadcherla et al., 2017; Yi et al., 2018). In addition, OPD may be the result of structural impairments because of an underlying genetic condition (Blake & Hudson, 2017). High-risk infants in South Africa may be prenatally exposed to alcohol and other substance abuse (De Beer, Kritzinger, & Zsilavecz, 2010; Okoroh et al., 2017). Exposure to the human immunodeficiency virus (HIV) and antiretroviral treatment are additional risks in lower-middle-income countries (LMICs) such as South Africa that may lead to subtle neurodevelopmental concerns influencing feeding (Salihu et al., 2012). Risk factors related to neonatal OPD are complex therefore they require further investigation (Cafferkey & Harrington, 2018).

The prevalence of OPD in neonates in intensive care is unknown (Hoogewerf, Horst, Groen, Niewenhuis, Bos, & Van Dijk, 2017; Schoeman & Kritzinger, 2017). Oropharyngeal dysphagia leads to nutritional compromise as a result of a feeding problem affecting the oral and/or pharyngeal stage of swallowing during neonatal admission (Viviers, 2016). It is estimated that 20% to 80% of infants discharged from intensive care may have experienced some degree of OPD during infancy (Jadcherla, 2016). A multidisciplinary team may assist with OPD management in high-risk infants (Schoeman & Kritzinger, 2017).

Treatment of OPD may involve many team members; however, it is the exclusive area of Speech-Language Therapists (SLTs) who assist in the evaluation of the underlying pathology and treatment of OPD (American Speech-Language-Hearing Association [ASHA], 2016; Piccione & Boesch, 2018). Understanding of the prevalence and risk profile associated with OPD in high-risk neonates in LMICs may lead to improved multidisciplinary teamwork, effective use of limited resources, enhancing early referral, and appropriate management before discharge (Jahan et al., 2018; Yi et al., 2018). The timely involvement of an SLT may greatly enhance the effectiveness of OPD management.

Currently, there is limited research in LMICs regarding the prevalence of neonatal OPD and associated factors of infants in Neonatal Intensive Care Units (NICUs) (Hoogewerf et al., 2017; Schoeman & Kritzinger, 2017). Most studies are conducted in high-income countries, where findings may not be generalisable to LMICs (McKinney et al., 2016). Neonatal OPD may be transient and vary in degree, where mild conditions are not reported, contributing to the challenge of identifying OPD (Viviers, Kritzinger, Vinck, & Graham, 2017). Thus, there is a need for better population-based data to gain an improved understanding of OPD in high-risk infants in neonatal care in LMICs (Jahan et al., 2018).

By determining the prevalence of OPD, the earliest intervention could occur before discharge, minimising long-term risks, and consequently saving lives. Human resources and effective intervention may then be prioritised in LMIC hospitals (Schoeman & Kritzinger, 2017). The aim of this study was to determine the prevalence of neonatal OPD at a given point in time, and to determine associated prenatal and perinatal risks of infants with this condition in neonatal care in a South African hospital.

## Study design

A cross-sectional observational study design was used to collect data prospectively for 1 month (Leedy & Ormrod, 2015).

### Setting

The research was conducted in the neonatal wards of an urban hospital in South Africa, where the hospital is awaiting final confirmation of Baby-Friendly Hospital Initiative-accreditation status. Expressed breast milk in a cup is the recommended alternative to direct breastfeeding in the hospital. Cup feeding is also given when mothers are not present. Oral feeding is initiated when infants weigh approximately 1500 g. Because of lack of space and high patient numbers, discharge may occur when infants only weigh approximately 1650 g and when oral feeding has been established. The hospital has 388 beds, with 57 neonatal intensive or high care beds divided between three wards, the Paediatric Intensive Care Unit, the neonatal ward, and the Kangaroo Mother Care (KMC) Ward.

### Study population

Participants were required to be medically stable and feeding orally for two days or longer. Neonates receiving enteral nutrition or who were not deemed medically stable for oral feeds by a paediatrician or neonatologist were excluded. A total of 126 participants were approached for participation in the study between 01 June 2018 and 30 June 2018. Eleven potential participants’ parents declined participation, three parents were not available to provide informed consent, and thirty-one participants were discharged before informed consent was obtained.

### Participants

Eighty-one participants were included in the sample (Table 1). Twenty-three participants were diagnosed with neurological conditions; however, the different conditions listed reflect the number of occurrences (see Table 1). The outstanding feature of the sample is the varied nature of the neonatal conditions with the majority of the participants presenting with multiple diagnoses or conditions.

**TABLE 1:** Participant characteristics (*N* = 81).

Infant characteristic	Minimum	Maximum	Mean	SD	*n*	%
Gestational age (weeks)	26	42	35.2	4.35	-	-
Chronological age (days)	2	69	11.7	15.65	-	-
Adjusted age at data collection (weeks)	28	42	36.46	3.26	-	-
Birth weight (grams)	845	4200	2263.2	859.25	-	-
Preterm birth (< 37 weeks)	-	-	-	-	47	58.0
Neonatal jaundice	-	-	-	-	28	34.6
Low birth weight (< 2500 g)	-	-	-	-	50	61.7
Very low birth weight (1001 g – 1500 g)	-	-	-	-	10	12.3
Extremely low birth weight (< 1000 g)	-	-	-	-	4	4.9
Respiratory distress	-	-	-	-	32	39.5
HIV exposure	-	-	-	-	13	16
Congenital pneumonia	-	-	-	-	11	13.6
Necrotising enterocolitis	-	-	-	-	10	12.3
Meconium exposed	-	-	-	-	15	18.5
**Neurological conditions**	-	-	-	-	**23**	**28.4**
Neonatal encephalopathy unspecified	-	-	-	-	12	14.8
Hypoxic-ischaemic encephalopathy	-	-	-	-	11	13.6
Bilateral periventricular echo density	-	-	-	-	6	7.4
Intraventricular haemorrhage	-	-	-	-	3	3.7
Meningitis	-	-	-	-	4	4.9
Neonatal convulsions	-	-	-	-	3	3.7
Hydrocephalus	-	-	-	-	1	1.2
Mild ventriculitis	-	-	-	-	1	1.2
Holoprosencephaly	-	-	-	-	1	1.2
Macrocephaly	-	-	-	-	1	1.2
Subarachnoid haemorrhage	-	-	-	-	1	1.2
**Chromosomal disorders**	-	-	-	-	**5**	**6.2**
Trisomy 21	-	-	-	-	3	3.7
Trisomy 13	-	-	-	-	1	1.2
Cornelia de Lange syndrome	-	-	-	-	1	1.2
**Other**	-	-	-	-	**4**	**4.9**
Amniotic band syndrome	-	-	-	-	1	1.2
Foetal alcohol spectrum disorder	-	-	-	-	1	1.2
Cleft lip	-	-	-	-	1	1.2
Prune belly syndrome	-	-	-	-	1	1.2

HIV, human immunodeficiency virus; SD, standard deviation.

The mean gestational age (GA) of participants was 35.2 weeks (standard deviation [SD] = 4.35), which is considered to be late preterm (World Health Organization [WHO], 2018). Participants on an average had LBW (< 2500 g), whereas the mean birth weight was 2263.2 g (SD = 859.25).

### Data collection

The Neonatal Feeding Assessment Scale (NFAS) (Viviers, Kritzinger, & Vinck, 2016) and a pulse oximeter (*Beurer PO30*) were used for data collection. The purpose of pulse oximeter measurements was to ensure that the physiological data had an objective component. Heart rate readings were used for the physiological subsystems subsection and oxygen saturation was monitored for safety reasons during the feeding assessment. The NFAS is a direct observational feeding assessment tool developed in South Africa. The tool is valid and developmentally supportive, and it is considered a minimally invasive method to identify possible OPD in high-risk infants in neonatal care (Viviers et al., 2016, 2017). The NFAS was previously validated against the gold standard, the modified-barium swallow study (MBSS) and it is shown to be reliable to detect OPD with acceptable inter-rater reliability (Viviers et al., 2017). The scale consists of six subsections, where pass or fail ratings for each section are recorded (Viviers et al., 2017) and a final diagnostic outcome is reached on which OPD identification is based. These subsections include the functioning of the: (A) physiological subsystems; (B) state of alertness during feeding; (C) stress cues during feeding; (D) general movement and muscle tone screening; (E) oral peripheral evaluation; and (F) clinical feeding and swallowing evaluation (Viviers, 2016). Oropharyngeal dysphagia was identified where participants received a minimum of three ‘Yes’ outcomes when scored on the six subsections of the NFAS, where at least one ‘Yes’ was obtained for the ‘oral peripheral evaluation’ or the ‘clinical feeding and swallowing evaluation’ categories. A risk assessment checklist (Kritzinger, 1994) was used to record demographic information and the collection of prenatal, perinatal, and postnatal data known to represent risks for OPD.

Participants’ feeding was clinically assessed once by the first author, an SLT registered with the Health Professions Council of South Africa (HPCSA) and trained in the procedures. Typically, neonatologists and nurses in the neonatal wards initiate the transition to oral feeds, where the infants who have difficulty with oral feeding are referred to an SLT for further assessment. Assessments were conducted during scheduled feeding times using the feeding method of choice, that is, breastfeeding or cup feeding. The NFAS was scored according to the guidelines, indicating whether participants were likely to present with OPD (Viviers, 2016). Inter-rater reliability was calculated. The researcher and second rater assessed the same feeding session of five participants and 100% agreement was obtained. The second rater had three years of clinical experience in assessment of paediatric dysphagia and intervention in the neonatal care setting, and is registered with the HPCSA as an independent practitioner. Participants’ medical files were perused and mothers were briefly interviewed for additional information on risks.

### Data analysis

The Statistical Package for the Social Sciences Version 25 software was used by a statistician to analyse data. Inter-rater reliability was confirmed. For continuous variables, descriptive statistics such as means and SDs were computed. For nominal categorical variables, frequency tables were created, and inferential statistics were computed, which included calculating phi coefficients and their corresponding *p*-values and odds ratios (ORs). A *p*-value of ≤ 0.05 was deemed statistically significant. The participants’ heart rate was scored using the pulse oximeter and subsection A of the NFAS, where a normal heart rate, tachycardia, or bradycardia was identified. The case history information obtained from the Risk Assessment was coded and analysed to determine associations between risks and OPD.

### Ethical considerations

Ethical approval (GW20180214HS) was obtained from the University of Pretoria, Faculty of Health Sciences Research Ethics Committee prior to commencing the study. Informed consent was obtained from the mothers on behalf of their infants.

## Results

### Prevalence of oropharyngeal dysphagia in the sample

For a diagnosis of OPD as per NFAS guidelines, each participant had to have a ‘Yes’ outcome for OPD for at least three subsections, where either subsection E or F was included (Table 2) to reach a final diagnostic outcome of OPD. Fifty-two participants (64.2%) presented with OPD because of a ‘Yes’ outcome for state of alertness (*n* = 41; 78.8%), stress cues (*n* = 52; 100%), oral peripheral evaluation (*n* = 48; 92.3%) and clinical feeding and swallowing evaluation (*n* = 52; 100%).

**TABLE 2:** Oropharyngeal dysphagia in the sample according to the Neonatal Feeding Assessment Scale outcomes (*n* = 52).

NFAS subsection	Dysphagia likely to be present	
	*n*	%
A. Physiological subsystems (heart rate and respiratory function)	27	51.9
B. State of alertness	41	78.8
C. Stress cues	52	100.0
D. General movement and muscle tone	25	48.1
E. Oral peripheral evaluation	48	92.3
F. Clinical feeding and swallowing evaluation	52	100.0

NFAS, Neonatal Feeding Assessment Scale.

Participants with OPD presented with a non-optimal state while feeding, where they were either asleep, drowsy, agitated or crying. They also presented with state stress cues or motor stress cues, and displayed mild, moderate or severe autonomic stress cues during oral feeding. Further characteristics of OPD included absent oral reflexes, asymmetrical appearance of their oral structures, where their oral musculature was either flaccid, inactive or stiff. One participant presented with a cleft lip and forty-four participants (84.62%) showed abnormal or limited movement of their oral structures. Participants with OPD also showed failure to initiate sucking, weak lip seal, weak or poor sucking, and reduced tongue movements during non-nutritive sucking or oral feeding (Section F).

Almost two-thirds of the sample of 81 neonates were identified as experiencing OPD (*n* = 52; 64.2%). Table 3 shows the prenatal and perinatal risks of the participants who presented with OPD (*n* = 52), and possible associations with OPD. Furthermore, Table 3 is an analysis based on the findings presented earlier in Table 1, where Table 3 illustrates only the participants with OPD (*n* = 52) and their associated prenatal and perinatal risks. Conditions in Table 1 that were not found in participants with OPD were removed from Table 3. Participants with OPD in Table 3 presented with more than one condition.

**TABLE 3:** Associations between prenatal and perinatal risk factors and the presence of oropharyngeal dysphagia (*n* = 52).

Risk factor	*n*	%	Phi coefficient	*p*-value (of Phi coefficient)	Odds ratio	*p*-value (of OR)
Breech presentation at birth	7	13.5	0.203	0.039	†	†
Septicaemia and other infections	14	26.9	0.241	0.030	4.974	0.044
Number of days on warm table/incubator: > 1 day	31	59.6	0.274	0.014	3.280	0.016
**Neurological conditions**	**19**	**36.5**	**0.242**	**0.030**	**3.598**	**0.036**
Neonatal encephalopathy	9	17.3	-	-	-	-
Hypoxic-ischaemic encephalopathy	8	15.4	-	-	-	-
Bilateral periventricular echo density	5	9.6	-	-	-	-
Intraventricular haemorrhage	3	5.8	-	-	-	-
Neonatal convulsions	2	3.8	-	-	-	-
Hydrocephalus	1	1.9	-	-	-	-
Meningitis	4	7.7	-	-	-	-
Holoprosencephaly	1	1.9	-	-	-	-
Macrocephaly	1	1.9	-	-	-	-
Subarachnoid haemorrhage	1	1.9	-	-	-	-

OR, odds ratio.

† , Odds ratio not calculable.

A significant association between participants with OPD and breech presentation at birth (*p* = 0.039) was found because of a positive phi coefficient. Septicaemia and other infections were significantly associated with participants with OPD (*p* = 0.030), where these participants were nearly five times more likely to have OPD (OR = 4.974). Participants who spent more than 1 day on a warm table or in an incubator was significantly associated with OPD (*p* = 0.014), and they were more than three times more likely to have OPD (OR = 3.280). Participants with neurological conditions were significantly more likely to have OPD (*p* = 0.030). These participants were three and a half times more likely to have OPD (OR = 3.598) than those without neurological conditions.

Frequently identified prenatal and perinatal risk factors in the group with OPD were further analysed for statistically significant associations with the subsections of the NFAS (Table 4). Only significant associations are presented.

**TABLE 4:** Statistically significant associations between prenatal and perinatal risks and Neonatal Feeding Assessment Scale subsections.

Risk factor	Subsection		*p*-value (of Phi coefficient)	Odds ratio	*p*-value (of OR)
	NFAS	Phi coefficient			
Prenatal exposure to smoking	A	0.270	0.015	†	†
Previous children: Mental disability, neurological disability, congenital disability	D	0.218	0.049	7.5	0.088
Preterm birth (<37 weeks)	B	0.223	0.044	2.593	0.047
Breech presentation at birth	A	0.238	0.032	5.543	0.049
Breech presentation at birth	E	0.224	0.044	†	†
Low birth weight (<2500 g)	B	0.282	0.011	3.378	0.013
Retinopathy of prematurity	A	0.238	0.032	5.543	0.049
Days on warm table and in incubator: >1 day	C	0.286	0.010	3.689	0.012
Neurological condition	E	0.285	0.010	5.051	0.016

NFAS, Neonatal Feeding Assessment Scale; OR, odds ratio.

† , Odds ratio not calculable.

Prenatal exposure to smoking (*p* = 0.015), breech presentation at birth (*p* = 0.032), and retinopathy of prematurity (*p* = 0.032) were significantly associated with OPD and likely to be related to *non-optimal physiological subsystems (A)*. Participants born breech (OR = 5.543), or who had retinopathy of prematurity (OR = 5.543) were almost six times more likely to present with OPD related to *non-optimal physiological subsystems*, respectively. Preterm birth (*p* = 0.044) and LBW (*p* = 0.011) were significantly associated with the presence of inadequate *state of alertness for feeding*. Participants who were preterm (OR = 2.593) or had an LBW (OR = 3.378) were around three times more likely to present with poor *state of alertness during feeding*. Participants who spent more than one day on a warm table or in an incubator was associated with displaying *stress cues* (*p* = 0.010). They were almost four times more likely to display OPD (OR = 3.689). Participants with OPD with siblings with mental disabilities, neurological disabilities or congenital disabilities were more likely to present with *poor general movement and muscle tone* (*p* = 0.049), contributing to their OPD. Participants who had siblings with disabilities were seven and a half times more likely to present with *poor general movement and muscle tone* (OR = 7.5). Participants who were born breech (*p* = 0.044) or had neurological conditions (*p* = 0.010) were significantly associated with a having poor outcome on the *oral peripheral examination*. Participants with neurological conditions (OR = 5.051) were five times more likely to present with a poor outcome on the *oral peripheral examination*. There were no specific prenatal or perinatal risk factors significantly associated with the NFAS subsection F, the *clinical feeding and swallowing performance* of the participants.

## Discussion

The study aimed to identify the prevalence of OPD and the associated prenatal, perinatal or postnatal risks for OPD in high-risk neonates at a single urban hospital in South Africa at a given point of time. An unexpectedly high prevalence of neonatal OPD of almost two-thirds (*n* = 52; 64.2%) of the sample (*n* = 81) was identified during one month, where the sample was deemed medically stable, ready to feed orally, and soon to be discharged. The prevalence of OPD in the general neonatal population is unknown, where studies typically focus on specific conditions (Jadcherla, 2016; Schoeman & Kritzinger, 2017). Such conditions include the following: Studies record a prevalence of 32.2% in late preterm infants compared with 7.4% in term infants, 3.41% in HIV exposed neonates, 30% in neonates after cardiac surgery, 16.9% in neonates with Neonatal Abstinence Syndrome, 12.1% in prenatal substance-exposed neonates, 26.5% in moderate to late preterm infants, and 37% of preterm infants in neonatal care (Engle, Tomashek, & Wallman, 2007; Hellmeyer et al., 2012; Mckean et al., 2017; Okoroh et al., 2017; Pike, Pike, Kritzinger, Krüger, & Viviers, 2016). It appears that a much higher prevalence of OPD is found when a study sample includes neonates with a wide range of medical conditions, and when all categories of preterm birth or LBW are considered. Prevalence data of OPD in the entire high-risk neonatal population in a specific facility enables resource planning and prioritisation of services. Further research focusing on the prevalence of OPD in the general neonatal population in different hospitals is required. The high prevalence of neonatal OPD in this study emphasises the importance of the SLTs’ involvement in neonatal care, and not only with those with severe or apparent feeding difficulty, as often occurs. SLTs should participate in decision-making about oral feeding readiness and coach parents during neonates’ transition from enteral to oral feeding in order to support and educate mothers before they are discharged and dispersed to communities with limited early intervention services.

The study aimed to identify risks associated with OPD. Infants born breech are at risk for low APGAR (Appearance, Pulse, Grimace, Activity and Respiration) scores, where respiratory assistance may be required (Fischbein & Freeze, 2018). In this study, prenatal exposure to smoking, breech presentation at birth, and retinopathy of prematurity were significantly associated with OPD, because of *non-optimal physiological sub-systems*. This may be the result of decreased oxygen saturation levels, increased heart rate, and increased apnoea events, which may consequently influence infants’ physiological stability necessary for oral feeding after birth (Kim et al., 2018).

Participants who spent more time in incubators were more likely to present with *stress cues* indicative of inadequate behavioural state for feeding. Participants may have displayed more stress cues because of environmental and nociceptive stimuli, such as painful medical interventions or separation from their caregivers (Crapnell, Rogers, Inder, Woodward, & Pineda, 2013; Viviers et al., 2016). A study by Pike et al. (2016) found no association between days spent in an incubator and OPD in preterm infants older than 32 weeks. The present study included extremely preterm infants, likely to spend longer periods in incubators possibly explaining this association.

Septicaemia and other infections may be considered to be a biological risk for OPD as a neonate’s length of hospitalisation may increase (Schoeman & Kritzinger, 2017). Infections may affect the central nervous system, which may increase risks for neurological complications and long-term sequelae, further negatively affecting oral feeding (March, Eastwood, Wright, Tilbrook, & Durrheim, 2014). A non-optimal behavioural state for oral feeding as a consequence of infection is known to contribute to feeding difficulties (Yi et al., 2018).

A significant association between neurological conditions, OPD, and a poor outcome on the *oral peripheral evaluation* of the NFAS was found. Participants with OPD showed poor reflexes and inappropriate oral structures for feeding. Infants with neurological conditions are likely to experience OPD because of impaired neural control required for the oral musculature and the coordination of sucking, swallowing, and breathing (Raol, Schrepfer, & Hartnick, 2018; Slattery, Morgan, & Douglas, 2012).

Participants with OPD who had siblings with mental or neurological disability or congenital disorders were more likely to present with *poor general movement and muscle tone*. The result could not be interpreted furthermore as it was not possible to establish which participants had the same conditions as their siblings.

It is known that preterm infants and those with LBW are at risk for OPD (Schoeman & Kritzinger, 2017), although no association was identified in this study. Neonates at the study hospital transition to oral feeds at a specific weight of 1500 g, regardless of their neurological maturity. A mean adjusted age of almost 37 weeks (36.46 weeks) at the time of assessment resulted in participants being considered as late preterm approaching discharge, where their initial feeding difficulties may have been transient, or may have resolved prior to the assessment. Therefore, the volume-driven feeding system (Shaker, 2017) employed by the hospital could explain why feeding difficulties were resolved. However, this study found an association between participants with preterm birth, LBW and *state of alertness*. Preterm or LBW participants often had a non-optimal state during feeding. It is known that preterm infants have inadequate state regulation that may affect oral feeding success (Lubbe, 2017). Preterm infants may be at risk for OPD because of respiratory difficulty and altered neurological development, further affecting their suck-, swallow- and breathe synchrony, and physiological state necessary for successful feeding (Shaker, 2017; Yi et al., 2018). As high-risk neonates have unique feeding profiles, cue-based or infant-driven feeding should be explored to tailor the introduction of oral feeding in the neonatal ward, although further research in this public health care hospital with resource restrictions and staff limitations is required (Shaker, 2017).

The high prevalence of OPD identified in the current study has several implications. Participants who were identified with OPD were referred to and managed by the SLT department at the study hospital. This led to an increase in patient load, resulting in high demands on the limited human resources in this department. A burden on the health care system may be because of neonates with OPD requiring longer hospitalisation and comprehensive multidisciplinary management (Mckean et al., 2017). If infants are treated early and appropriately, neonatal OPD may be resolved or managed quickly, leading to less pressure on the health system. High patient turnover in the study hospital may result in limited time for nurses to feed neonates and coach parents effectively. Interprofessional collaboration between SLTs, nurses, neonatologists, and dieticians should be the standard of care, where skills are shared, and preparation for feeding readiness is coordinated and comprehensive. An example of an effective program for neonatal OPD associated with LBW and preterm birth is the premature infant oral motor intervention (PIOMI) (Ghomi et al., 2019).

High-risk neonates could be monitored by SLTs in an infant dysphagia clinic after discharge to ensure OPD is managed appropriately or to prevent adverse outcomes. The earliest involvement of the SLT may lead to specific parent coaching prior to discharge, where parents are supported in detecting and interpreting infants’ cues and adjusting their handling to optimise feeding (Kritzinger & Van Rooyen, 2014). Kangaroo Mother Care may be a viable option to improve parental independence and breastfeeding outcomes in high-risk infants (Shrivastava, Shrivastava, & Ramsamy, 2013). Because of the complex nature of OPD, diagnosis and management may be difficult, especially, in LMICs where instrumental assessment is not readily available (Jadcherla et al., 2017). Although MBSS remains the ideal to assess swallowing in infants, the NFAS may assist in identifying whether OPD is likely to be present in resource-limited settings (Viviers et al., 2016). Future research with larger samples in similar settings and utilising a longitudinal design, may be of value.

## Conclusion

This study is the first of its kind to document the prevalence of neonatal OPD in a specific lower-middle-income setting and found that 64.2% of the 81 participants presented with OPD. This rate was unexpectedly high as participants were mostly approaching discharge and were deemed medically stable to commence oral feeding. However, 31 neonates were discharged before informed consent could be requested for data collection. This observation and the fact that neonates are often discharged from this hospital at only 1650 g, may equate to neonates possibly leaving neonatal care with unidentified, or mild OPD, because of limited beds and high patient-volumes. Oropharyngeal dysphagia may be treated and adverse sequelae of OPD prevented with the earliest involvement of the SLT in neonatal units. The SLT has unique access to neonates and mothers during their hospitalisation before they are discharged and dispersed to communities without early intervention services. Future research may be directed towards the general high-risk neonatal population and the prevalence of neonatal OPD in similar settings.
